# A Data-Driven Investigation on Surface Electromyography Based Clinical Assessment in Chronic Stroke

**DOI:** 10.3389/fnbot.2021.648855

**Published:** 2021-07-15

**Authors:** Fuqiang Ye, Bibo Yang, Chingyi Nam, Yunong Xie, Fei Chen, Xiaoling Hu

**Affiliations:** ^1^Department of Biomedical Engineering, The Hong Kong Polytechnic University, Hong Kong, China; ^2^Department of Electrical and Electronic Engineering, Southern University of Science and Technology, Shenzhen, China

**Keywords:** chronic stroke, clinical assessment, surface electromyography, data-driven model, upper limb

## Abstract

**Background:** Surface electromyography (sEMG) based robot-assisted rehabilitation systems have been adopted for chronic stroke survivors to regain upper limb motor function. However, the evaluation of rehabilitation effects during robot-assisted intervention relies on traditional manual assessments. This study aimed to develop a novel sEMG data-driven model for automated assessment.

**Method:** A data-driven model based on a three-layer backpropagation neural network (BPNN) was constructed to map sEMG data to two widely used clinical scales, i.e., the Fugl–Meyer Assessment (FMA) and the Modified Ashworth Scale (MAS). Twenty-nine stroke participants were recruited in a 20-session sEMG-driven robot-assisted upper limb rehabilitation, which consisted of hand reaching and withdrawing tasks. The sEMG signals from four muscles in the paretic upper limbs, i.e., biceps brachii (BIC), triceps brachii (TRI), flexor digitorum (FD), and extensor digitorum (ED), were recorded before and after the intervention. Meanwhile, the corresponding clinical scales of FMA and MAS were measured manually by a blinded assessor. The sEMG features including Mean Absolute Value (MAV), Zero Crossing (ZC), Slope Sign Change (SSC), Root Mean Square (RMS), and Wavelength (WL) were adopted as the inputs to the data-driven model. The mapped clinical scores from the data-driven model were compared with the manual scores by Pearson correlation.

**Results:** The BPNN, with 15 nodes in the hidden layer and sEMG features, i.e., MAV, ZC, SSC, and RMS, as the inputs to the model, was established to achieve the best mapping performance with significant correlations (*r* > 0.9, *P* < 0.001), according to the FMA. Significant correlations were also obtained between the mapped and manual FMA subscores, i.e., FMA-wrist/hand and FMA-shoulder/elbow, before and after the intervention (*r* > 0.9, *P* < 0.001). Significant correlations (*P* < 0.001) between the mapped and manual scores of MASs were achieved, with the correlation coefficients *r* = 0.91 at the fingers, 0.88 at the wrist, and 0.91 at the elbow after the intervention.

**Conclusion:** An sEMG data-driven BPNN model was successfully developed. It could evaluate upper limb motor functions in chronic stroke and have potential application in automated assessment in post-stroke rehabilitation, once validated with large sample sizes.

**Clinical Trial Registration:** www.ClinicalTrials.gov, identifier: NCT02117089.

## Introduction

Stroke is one of the leading causes of upper limb disability and affects about 15 million individuals worldwide annually (Langhorne et al., 2011). Around 65 percent of survivors with chronic stroke, i.e., 6 months after the onset of a stroke, are not able to use their affected hands for daily tasks (Dobkin, 2005). Repetitive voluntary, high-intensity practice with the paretic upper limb could accelerate the recovery of motor function after stroke (Harris and Eng, 2010). However, conventional rehabilitation services are usually “one-to-one” manual operations, which are not able to cope with the rapidly growing chronic stroke population (Woo et al., 2008).

Robot-assisted rehabilitation systems could reduce the manpower requirement for professional therapists by providing repetitive and intensive training and services for the growing stroke population in the long term (Norouzi-Gheidari et al., 2012). Many robotic systems for upper limb rehabilitation have been developed and proven feasible and effective for restoring upper limb functions, e.g., HapticKnob (Lambercy et al., 2011), Haptic Master (Timmermans et al., 2014), and Robotic hands (Nam et al., 2017). Voluntary effort-based robot-assisted stroke rehabilitation systems recruit the active movement from residual neuromuscular pathways and lead to better motor recovery and longer sustainability than passive robot-assisted systems without voluntary efforts (Volpe et al., 2005; Hu et al., 2009b). Surface electromyography (sEMG) can represent voluntary effort with a resolution of individual muscular activities; thus, sEMG-triggered control has been widely adopted in stroke rehabilitation robots, to maximize the involvement of voluntary efforts during post-stroke training (Hu et al., 2012; Basteris et al., 2014). In our previous studies, a series of sEMG-driven robot-assisted systems have been developed for chronic stroke rehabilitation, which significantly improved motor recovery of the upper limb (Hu et al., 2013; Nam et al., 2017).

To evaluate rehabilitation training effects, clinical assessments must be performed before and after rehabilitation interventions. For example, the Fugl–Meyer Assessment (FMA) (Fugl-Meyer et al., 1975), the Motors Status Scale (MSS) (Aisen et al., 1995), the Action Research Arm Test (ARAT) (Lyle, 1981), and the Modified Ashworth Scale (MAS) (Ashworth, 1964) were frequently used for activity or body function evaluation in upper limb rehabilitation training (Wei et al., 2011). Among them, FMA and MAS have been widely adopted as the clinical assessments of motor functional improvement and muscular spasticity changes (Coote et al., 2008; Hu et al., 2009a; Wei et al., 2011). The clinical assessments are still the “golden standards” for measuring the effects of stroke rehabilitation interventions (Simbaña et al., 2019). However, most clinical assessments are still manually conducted by therapists and rely heavily on manual operations. Manual clinical assessments not only require considerable manpower but are also time-consuming and costly. An automated rehabilitation process based on a robotic system also offers a feasible solution to the increased need for clinical assessments to control the quality of rehabilitation. Taking these factors into account, there is a demand for quantitative assessments to evaluate the efficacy of long-term robot-assisted rehabilitation for chronic stroke survivors.

Quantitative assessment using sEMG data emerged as a novel approach to monitoring motor recovery. sEMG has been widely used for investigating pathophysiology (Li et al., 2006), monitoring neuromuscular progressive change (Hu et al., 2009a; Li et al., 2014), estimating muscle force (Xu et al., 2018), and detecting muscle fatigue (Campanini et al., 2020). In our previous studies, motor recovery during sEMG-driven robot-assisted rehabilitation was evaluated according to the quantitative sEMG parameters, i.e., the activation level and co-contraction index (CI), which corresponded to muscle spasticity and co-activation patterns, respectively (Hu et al., 2009a, 2012, 2013; Nam et al., 2017; Qian et al., 2017; Huang et al., 2020). Another study proposed the use of fuzzy approximate entropy to investigate the complexity of sEMG signals to monitor the motor recovery during the robot-assisted rehabilitation (Sun et al., 2013). These studies indicated that sEMG data could be employed for quantitative and automated assessment of rehabilitation effects during sEMG-driven robot-assisted rehabilitation intervention for chronic stroke survivors. However, these studies only calculated the mathematical parameters from sEMG data to assess motor recovery and did not map sEMG data to clinical scales, which were largely confined to clinical applications for therapists. Moreover, interpretation of sEMG data has been identified as a technical barrier for both clinical professionals and technologists to use sEMG in automated assessments (Merletti et al., 2021).

Alternatively, automated assessments can reduce manpower requirements, produce fast measures of motor recovery automatically, assist in diagnosis, and enable customization of therapies. One promising technique for automated assessment is machine learning, which has been applied to determine the relationship between biomarkers extracted from bioinformatics data and the corresponding clinical scales (Otten et al., 2015; Yu et al., 2016; Wang C. et al., 2020). In the previous studies, multi-modality fusion systems were developed to combine both kinematic data and muscular characteristic data, i.e., sEMG data, to quantitatively assess upper limb motor function for post-stroke rehabilitation, and they revealed significant correlations between the automated assessment results and the standard clinical scores (Zhang et al., 2019; Wang C. et al., 2020). However, the results of the multi-modality fusion systems used a modified outcome scale, which was different from FMA scores and not accepted by clinicians without further clinical validation (Simbaña et al., 2019). Furthermore, the kinematic data-driven automated assessments required extra devices for recording kinematic data in human movement, which were complicated for therapists to operate (Otten et al., 2015; Yu et al., 2016; Wang C. et al., 2020). As a result, they did not save much manpower, cost, and time, compared to manual assessments. Recently, a novel spasticity evaluation method has been proposed to map sEMG data to the MAS with an adaptive neuro fuzzy inference system, which achieved a high accuracy between the regression results and manual MAS scores (Yu et al., 2020). However, this automated assessment only focused on the quantitative evaluation of spasticity without investigating voluntary functional recovery, which was usually evaluated by FMA in clinical assessments. In addition, this quantitative spasticity evaluation approach required repetitive passive stretches conducted by therapists to acquire sEMG data, which meant that it was not suitable for automated assessment of motor recovery. For mapping sEMG data to clinical scales, although multiple linear regression model is explainable for multi-factor regression analysis, backpropagation neural network (BPNN) is more suitable for analyzing sEMG signals because of a higher compatibility than the linear regression analysis when input data have large varieties, e.g., normality, linearity, extremities, and missing values (Uyanik and Güler, 2013). Moreover, the BPNN has been found to outperform the multiple regression model in the non-linear regression tasks (Tu, 1996). Specifically, the BPNN has been employed to work out regression mapping issues related to sEMG data, e.g., estimation of the joint angle (Aung and Al-Jumaily, 2013; Yang et al., 2019) and sketching pattern recognition (Yang and Chen, 2016). These studies indicated that the BPNN had the potential to map sEMG data to clinical scales. To the best of our knowledge, no research has investigated the problem of mapping sEMG data to the clinical scales of both FMA and MAS for automated assessment in sEMG-driven robot-assisted rehabilitation.

In summary, the purpose of this study was to design a novel sEMG data-driven model for mapping sEMG data to two widely used clinical scales, i.e., FMA and MAS, during a robot-assisted rehabilitation for chronic stroke survivors. The rest of the paper is organized as follows. In Methodology, the framework and configurations of the data-driven model are introduced. The experimental results are described in results, and discussions are presented in discussion. Finally, conclusion concludes the work.

## Methodology

In this work, participants with chronic stroke were recruited to receive a 20-session sEMG-driven robotic-hand-assisted intervention in a neurorehabilitation laboratory at Hong Kong Polytechnic University. Evaluations by sEMG and clinical assessments were conducted before and after the intervention. A data-driven model was established by using the sEMG signals and clinical scales as datasets to investigate the mapping relationship between the sEMG signals and the clinical scales.

### Subject Recruitment

After this work obtained ethical approval from the Human Subjects Ethics Committee of Hong Kong Polytechnic University (HSEARS20130306005-04 and CRE-2013.283-T), 29 hemiplegic participants with chronic stroke were recruited from local districts according to the following inclusion criteria: (1) ages ranging from 18 to 78 years, (2) at least 6 months after the diagnosis of a singular and unilateral brain lesion due to stroke, (3) able to extend the metacarpophalangeal and interphalangeal joints of the fingers to 170° passively, (4) MAS ≤ 3 for spasticity at the finger, wrist, and elbow joints during extension, (5) moderate to severe motor impairments in the affected upper limb as assessed by the FMA (15 < FMA < 45), (6) without cognition impairments as assessed by the Mini-Mental State Examination (MMSE) > 21 (Folstein et al., 1975), and (7) detectable voluntary sEMG signals (three times of the standard deviations (SD) above the sEMG baseline) from the target muscles of extensor digitorum (ED), flexor digitorum (FD), biceps brachii (BIC) and triceps brachii (TRI) on the affected side. The demographic data of the participants are shown in Table 1. Written informed consents had been signed by participants prior to the inclusion in this study.

**Table 1:** Demographic characteristics of the participants with chronic stroke.

**Participant no**.	**Gender (female/male)**	**Stroke type (hemorrhagic/Ischemia)**	**Side of hemiparesis (left/right)**	**Age (years) mean±SD**	**Years after onset of stroke** **mean ± SD**
29	6/23	12/17	17/12	58.7 ± 8.3	7.1 ± 4.0

### sEMG-Driven Robotic-Hand-Assisted Intervention

**Figure 1:**
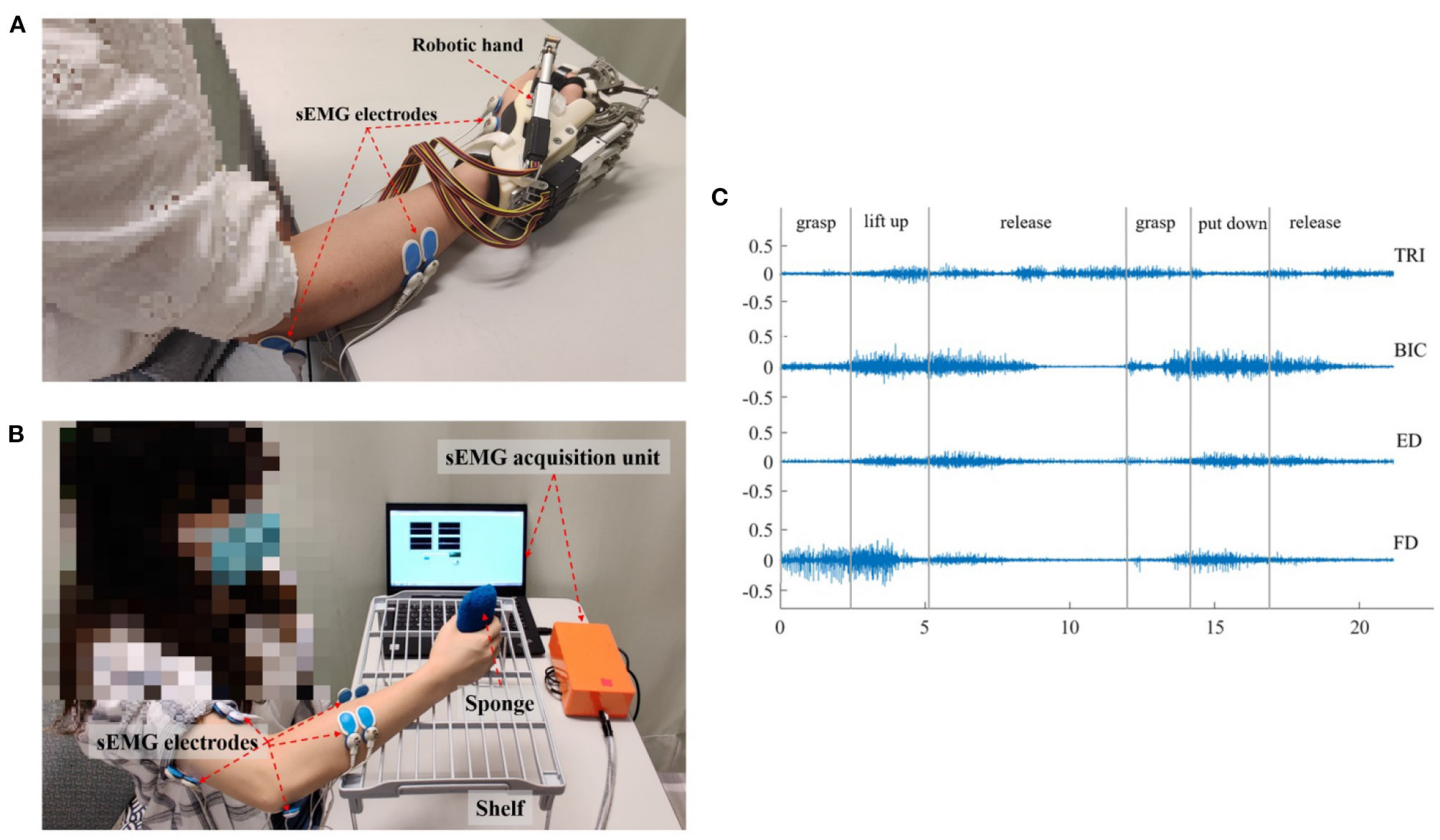
The experimental setup and representative sEMG signals. **(A)** The sEMG-driven robotic hand system. **(B)** The setup for data acquisition during the vertical bare hand evaluation task. **(C)** The representative raw sEMG trials for the four muscles of TRI, BIC, ED, and FD in a vertical bare hand evaluation task. Each movement during the evaluation task was manually marked by the experiment operator.

The sEMG-driven robotic-hand system used in this study is shown in Figure 1A. This robotic hand can provide mechanical assistance to finger extension and flexion of the affected upper limb for stroke survivors. sEMG-triggered control was used in this study. The sEMG signals from the abductor pollicis brevis (APB) and ED muscles were used as voluntary neural drivers to initiate the robot assistance for phasic and sequential limb tasks, i.e., hand closing and hand opening. Once the robotic assistance was started, no muscular effort was required of the user, i.e., sEMG-triggered mode. Three times of the SD above the sEMG baseline during the resting state was preset as the threshold level to trigger the mechanical assistance. More detailed information about the control mechanism can be found in our previous studies (Hu et al., 2013; Nam et al., 2017; Huang et al., 2020). All participants received the sEMG-driven robotic-hand-assisted intervention, consisting of 20 intervention sessions at a frequency of 3–5 sessions per week within 7 consecutive weeks. During each intervention session, participants were required to perform 30-min lateral and 30-min vertical arm reaching and grasping tasks, with a 10-min interval between the two tasks to avoid muscle fatigue, as described elsewhere (Nam et al., 2017). All sEMG signals were captured by sEMG electrodes (Blue Sensor N, Ambu Inc. with a contact size of 20 × 30 mm) at a sampling rate of 1000 Hz (DAQ, 6218 NI DAQ card; National Instruments Corp.) Then, they were amplified 1000-fold using a preamplifier (INA 333; Texas Instruments Inc., Dallas, TX, USA) to calculate the real-time sEMG levels of the driving muscles for triggering the mechanical assistance of the robotic hand (Hu et al., 2013). As shown in Figure 1A, sEMG electrode pairs (inter distance of 20 mm) were attached to the skin with the orientation parallel to the muscle fibers, according to the configuration specified in SENIAM guideline (Hermens et al., 1999).

### Clinical Assessment

Each participant was clinically assessed with the FMA and MAS, before and after the 20-session interventions, i.e., pre-intervention and post-intervention sessions. FMA was treated as the primary outcome and MAS was regarded as a secondary outcome in this work, as practiced in the literatures (Krebs and Volpe, 2013; Otten et al., 2015). The FMA was widely adopted as the primary assessment measure in clinical trials of stroke rehabilitation, because of its high responsiveness in capturing levels of motor functional impairment in the upper limb (Fugl-Meyer et al., 1975; Wei et al., 2011). The FMA upper limb assessment had a total score of 66, which could be further divided into shoulder/elbow scores (i.e., FMA-SE, 42/66) and wrist/hand scores (i.e., FMA-WH, 24/66) (Krebs and Volpe, 2013) for more detailed measures of distal and proximal paretic upper extremity movement (Page et al., 2012). Meanwhile, MAS was used as an assessment independent from the FMA, to measure muscular spasticity related to involuntary muscle contraction after stroke (Wei et al., 2011). MASs for the finger, wrist, and elbow were measured in passive joint extension and flexion manually by a professional clinically, grading at 0, 1, 1+, 2, 3, and 4, where 1+ was numerically represented as 1.4 in this study, as practiced previously (Wei et al., 2011; Qian et al., 2019). The two clinical assessments were measured manually by a blinded assessor without knowledge of the research purpose and details of the study.

### sEMG Assessments in Reaching Tasks

Besides the FMA and MAS clinical assessments, sEMG signals in reaching tasks, i.e., the bare hand evaluation tasks of the paretic upper limb, were captured as the objective assessment before and after the 20-session intervention (Hu et al., 2013). Four target muscles related to the bare hand evaluation task were investigated, i.e., ED, FD, BIC, and TRI, which corresponded to finger extension, finger flexion, elbow flexion, and elbow extension, respectively, during the reaching task. Compensatory muscular activities among the four target muscles were expected during the bare hand evaluation task for stroke survivors, since proximal compensation is commonly observed in chronic stroke (Levin et al., 2009; Bakhti et al., 2018; Qian et al., 2019). The sEMG signals of the four muscles were used as the inputs to the data-driven model through supervised machine learning, which built the mapping relationship between the sEMG signals and clinical scales.

In each sEMG assessment session, the participant was seated in front of a table with a vertical distance of 30–40 cm between the surface of the table and the participant's shoulder (Figure 1B). Then, the participant was instructed to perform a bare hand evaluation task while voluntary muscle contraction was monitored. In the real-time recording, an active bandpass filter circuit was designed with the center frequency of 118 Hz, the high-pass cutoff frequency at 2 Hz, and the low-pass cutoff frequency at 7k Hz (Nam et al., 2017). sEMG signals were collected from the target muscles (ED, FD, BIC, and TRI) of the affected upper limb during the motion, with a sampling frequency of 1000 Hz (DAQ USB-6009), and the sampling accuracy of the DAQ was 14 bits. In the evaluation task, each participant was instructed to grasp a sponge, with a thickness of 5 cm and a weight of 30 g, place it at the midline of the lower layer of a shelf, lift it through a vertical distance of 17 cm, and put it on the midline of the upper layer of the shelf. Then, the participant was required to release the sponge, grasp it again, and put it back in the initial location. The bare hand evaluation task was conducted at the natural speed of the participant and was repeated three times with a 2-min break to avoid muscle fatigue. The sEMG signal in a trial was recorded when the participant began to grasp the sponge, i.e., once their fingers touched the sponge, and was stopped when the participant released the sponge at the initial point, i.e., all fingers left the sponge, all while being monitored by an experimental operator. Early studies showed that most people with chronic stroke could grasp the sponge but could not release it because of muscle spasticity (Hu et al., 2013). Thus, a maximum time limit of 10 s was set, i.e., participants were allowed to use the unaffected hand to take off the sponge if their paretic hand could not release it within 10 s. Pauses and repetitions during the evaluation were allowed, particularly for the pre-intervention assessment, since most of the participants had weakness and muscular discoordination in the affected upper limb. The pauses in the trials were marked by the operator during the recording, and the sEMG episodes associated with the pauses were removed in offline processing. Once the participant's fingers left the sponge, the operator marked the pause. When the participant grasped the sponge again, the operator marked the end of pause. Figure 1C shows the representative sEMG signals in the vertical bare hand evaluation task.

### Data Preparation

After the removal of the pauses in the evaluation, the average duration of the sEMG trials was 30.92 s (±9.93 s, standard deviation), with a minimum of 9.8 s and a maximum of 51.8 s. The reason for the relatively large variation in the signal lengths was that some participants (*n* = 9) took less time to perform the evaluation task after receiving the intervention, while some participants did not improve the speed of movements.

Before feeding the sEMG signals into the data-driven model, the digitized sEMG signals were filtered by a 4th-order Butterworth band-pass filter of 10–500 Hz and a notch filter of 50 Hz in offline processing (Matlab 2017b, MathWorks Inc.). The sEMG signals were subjected to the investigation of the mapping relationship with (1) the FMA subscores of the shoulder/elbow (FMA-SE) and the wrist/hand (FMA-WH), and (2) MASs for the elbow, wrist, and finger joints. For the mapping to MAS scores, the sEMG signals were further low-pass filtered (4th-order Butterworth filter) with different cutoff frequencies of 80, 150, 200, 300, 400, and 500 Hz. This was done in order to evaluate the mapping performance with an effective frequency domain, which would capture the features mainly related to slow involuntary contractures in a spastic muscle after stroke (Dromerick, 2002).

In the offline sEMG processing, a trial of sEMG was segmented into epochs, with a length of 400 ms overlapped by 200 ms as in (Atzori and Müller, 2019), to achieve a balance between the necessary sEMG information for analysis of the myo-states and stationarity of the signal epochs. Each 400-ms sEMG epoch was verified to be wide-sense stationary, i.e., its mean and autocorrelation function were time-invariant (Yates and Goodman, 1999). Subsequent feature extraction and investigation of the mapping relationship between the sEMG signals and clinical scales, i.e., FMA-WH, FMA-SE, and MASs for the finger, wrist, and elbow, were based on the segmented sEMG signals.

**Figure 2:**
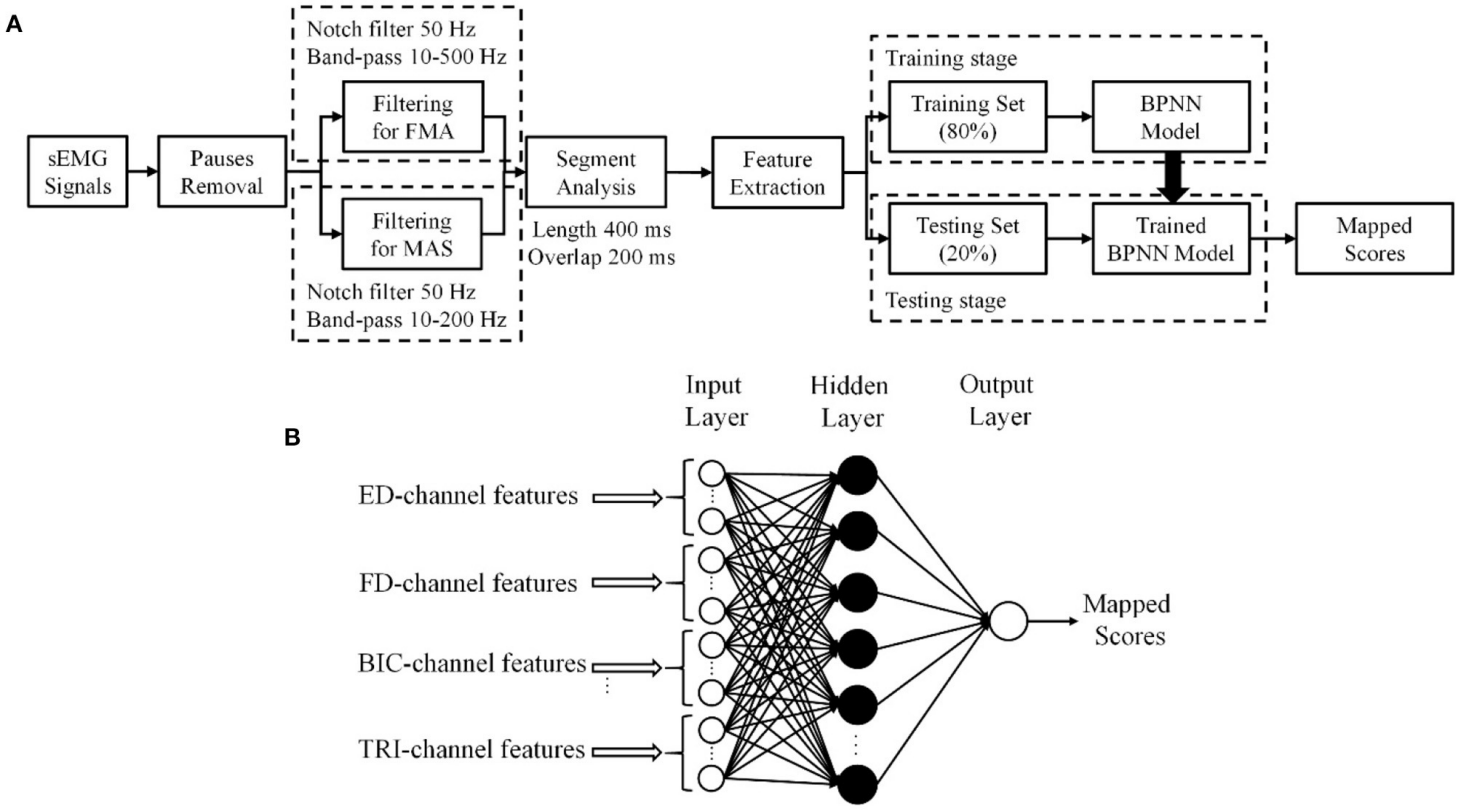
**(A)** The procedure of sEMG signal processing. **(B)** The structure of the three-layer backpropagation neural network (BPNN) data-driven model.

Then, eighty percent of the sEMG epochs in a trial were used as the training data and the remaining 20% were adopted as the testing data, according to the Pareto principle (Dobbin and Simon, 2011; Ramesh et al., 2019). The sEMG epochs from the same sEMG trial of a participant would be mapped to the clinical scores collected from the participant in the related evaluation session, i.e., either pre- or post-intervention. Five-fold cross validation was used to ensure that each sEMG epoch in a trial was fully utilized in the testing stage and the average of the performance of the testing data over the 5 folds represented the overall model performance (Mostafavi et al., 2015). The overall flowchart of the sEMG signal preparation with the data-driven model is shown in Figure 2A.

### The sEMG Based Data-Driven Model

An sEMG based data-driven model was established based on the BPNN according to the following steps: (1) sEMG feature extraction, (2) setup of the BPNN model, (3) configuration of the hidden layer, and (4) sEMG feature selection.

#### sEMG Feature Extraction

Abstracted features of sEMG signals in the time domain have been adopted for the recognition of dynamic myo-activities (Tsai et al., 2014; Nazmi et al., 2016). There were five widely adopted features, i.e., root mean square (RMS), slope sign change (SSC), mean absolute value (MAV), zero crossing (ZC), and wavelength (WL) (Hudgins et al., 1993). MAV, RMS, and WL were related mainly to the intensity of sEMG magnitude. The magnitude of sEMG signals changed as muscle contraction levels vary in real time (Tsai et al., 2014), and was commonly assumed to be proportional to muscle force (Hof and Van Den Berg, 1981). SSC and ZC mainly reflected the neuromuscular dynamics in the frequency domain and were related to motor unit (MU) firing properties (Nazmi et al., 2016; Wang K. L. et al., 2020). The mathematical expressions for the features are described by the following formulas, where x(t) is the time series in one epoch, and T is the number of samples in one epoch (i.e., T = 400).

RMS mainly reflects the absolute magnitude of the signal, given as:

(1) $$RMS\left(x\right)=\sqrt{\frac{1}{T}\sum_{t=1}^{T}x^{2}(t)}.$$

MAV is calculated by taking the average of the absolute value of sEMG signals *x*(*t*), as follows:

(2) $$MAV\left(x\right)=\frac{1}{T}\sum_{t=1}^{T}\left|x(t)\right|.$$

ZC is the number of times that the amplitude value of sEMG signals crosses the zero axis, formulated as:

(3) $$\{\begin{array}{c} ZC\left(x\right)=\sum_{t=1}^{T-1}\left(\left(x\left(t+1\right)\times x\left(t\right)\right)\cap \left|x\left(t\right)-x\left(t+1\right)\geq 0\right|\right)\\ sign\;\left(x\right)=\{\begin{array}{c} 1,if\;x\geq 0\\ 0,\;else\; \end{array} \end{array}$$

SSC measures the number of times the sign changes in the slope of the sEMG signals, as follows:

(4) $$\{\begin{array}{c} SSC\left(x\right)=\sum_{t=2}^{T-1}f(\left(x\left(t\right)-x\left(t-1\right)\right)\times \left(x\left(t\right)-x\left(t+1\right)\right))\\ f\left(x\right)=\{\begin{array}{c} 1,\;if\;x\geq 0\\ 0,\;else\; \end{array} \end{array}$$

WL is the cumulative measure of the length of the signal, given as:

(5) $$WL=\frac{1}{T}\sum_{t=1}^{T-1}\left|x\left(t+1\right)-x(t)\right|.$$

The five features extracted from the segmented sEMG signals of the target muscles formed the input vectors to the BPNN whose output would be the mapped scores.

#### Setup of the BPNN Model

A three-layer, i.e., one input layer, one hidden layer, and one output layer, BPNN was established as the data-driven model to investigate the mapping relationship between the extracted sEMG features and the clinical scales (Figure 2B).

The essence of BPNN was to minimize the error of the network outputs with the derivatives of the error function (Hecht-Nielsen, 1992), where the weight factors were updated by iterative backpropagation. In each iteration, the sEMG input vectors were arranged for the network, i.e., ${\overset{⇀}{X}}_{k}=\left[{\overset{⇀}{X_{\;}}}_{ED,\;k},\;{\overset{⇀}{X_{\;}}}_{FD,\;k},\;{\overset{⇀}{X_{\;}}}_{BIC,k},\;{\overset{⇀}{X_{\;}}}_{TRI,\;k}\;\right],$ where *k* represented the *k*^*th*^ sEMG epoch from the four muscles, and ${\overset{⇀}{X_{\;}}}_{m,k}$ was the sEMG feature vector with the elements obtained from Equation 1–5 for the muscle *m*. The projection for the mapping from the feature vectors to the output clinical scales can be described by

(6) $$f\left(x\right)=W\cdot {\vec{X}}_{k}+b,$$

where *W* is the respective weight matrix and *b* is the bias vector of the network. *W* and *b* were updated iteratively by the calculation of the error function with the BP algorithm (Lecun et al., 1998). In this work, the sigmoid function was used as the activation function, and Bayesian regularization was employed to train the network. The outputs, i.e., the mapped scores, of the BPNN are continuous values with decimals, while the manual measurement scores are integral numbers, because of the adopted sigmoid function and gradient correction of the BP algorithm. The mean value of the outputs for all sEMG epochs in the testing set from a participant was regarded as a final mapped score. The accuracy of the mapped results was assessed by the Pearson correlation of the results with the manual clinical scales. The significance level of the correlation was set at 0.05. The significance at levels 0.01 and 0.001 are also indicated.

#### Configuration of the Hidden Layer

In the configuration of the hidden layer structure, the correlation strength between the mapped scores and the manual clinical scales, as indicated by the Pearson correlation coefficient, *r*, were evaluated with different numbers of neurons, or nodes in the hidden layer according to the two-phase method (Karsoliya, 2012). A very strong correlation (Evans, 1996), i.e., *r* > 0.9, with a concise number of hidden nodes was adopted in the configuration. The input layer contained 20 nodes from the 4-channel muscles (i.e., 5 parameters were extracted from sEMG signals of each muscle channel). Then, the BPNN models with 10, 15, 20, 30, 40, 50, 100, 150, and 200 nodes in the hidden layer were trained by the 80% sEMG epochs and the corresponding FMA-WH and FMA-SE manual scores, both pre- and post-intervention sessions. The models were fed with the remaining 20% sEMG epochs during the testing stage, whose outputs were assessed for correlation performance with the manual scores in the testing set. FMA was employed in the model configuration because it was the primary outcome of the sEMG-driven robot-assisted intervention in this work. Table 2 shows the correlation coefficients, *r*, between the FMA scores and the mapped scores obtained using the BPNN model with different numbers of nodes in the hidden layer. A three-layer BPNN with 15 nodes in the hidden layer was used in this study, yielding strong correlations above 0.9 for both FMA-SE and FMA-WH (see Table 2 for a detailed description in the Results section). Bayesian regularization was employed in the training stage to avoid the overfitting problem (Burden and Winkler, 2008), which could be caused by the redundant hidden nodes.

**Table 2:** The correlation coefficients, *r*, between the mapped and manual FMA scores obtained by the BPNN model with different numbers of nodes in the hidden layer.

**Number of nodes in hidden layer**	***r* with FMA-SE**	***r* with FMA-WH**
10	0.89^***^	0.91^***^
15	0.90^***^	0.93^***^
20	0.88^***^	0.91^***^
30	0.86^***^	0.89^***^
40	0.88^***^	0.87^***^
50	0.89^***^	0.86^***^
100	0.85^***^	0.86^***^
150	0.84^***^	0.85^***^
200	0.82^***^	0.90^***^

Significance levels are indicated as * for P ≤ 0.05, ** for P ≤ 0.01, and

*** *for P ≤ 0.001*.

*The bold values are the highest correlation coefficients achieved*.

#### sEMG Feature Selection

Then, based on the developed BPNN stated above, a selection of optimal feature vectors was performed after the sEMG feature extraction, with the purpose of reducing the dimensions of the input to the BPNN model (Phinyomark et al., 2012). The five features, i.e., MAV, RMS, WL, SSC, and ZC, were divided into two groups. Group I included MAV, RMS, and WL, which were related to the magnitude of the sEMG signals; group II included ZC and SSC, which mainly reflected the MU firing statistics. Different combinations of the features from the respective two groups, i.e., at least one feature from each group, were selected according to Table 3 as the input vectors to the BPNN, to investigate the correlation between the mapped scores and the manual FMA-WH and FMA-SE scores. The combination, including both the time domain magnitude-related and the MU-firing-related sEMG features, was to search for a set of relatively complete neuromuscular features with low redundancy. Bayesian regularization was employed to avoid the overfitting problem (Burden and Winkler, 2008), in case of the potential redundancy introduced by the reduction of input nodes. The feature combination of MAV, SSC, RMS, and ZC was selected, because this had the highest correlation coefficients with both FMA-WH and FMA-SE, as shown in Table 3. A detailed description of Table 3 is given in the Results section.

**Table 3:** The correlation coefficients, *r*, between the mapped and manual FMA scores obtained by different sEMG feature combinations.

**Featurecombinations**	***r* with FMA-SE**	***r* with FMA-WH**	**Feature combinations**	***r* with FMA-SE**	***r* with FMA-WH**
MAV	0.80^***^	0.61^***^	MAV+WL+SSC	0.86^***^	0.65^**^
ZC	0.78^***^	0.82^***^	MAV+WL+ZC	0.79^***^	0.88^***^
SSC	0.73^***^	0.73^***^	RMS+WL+SSC	0.88^***^	0.86^***^
RMS	0.71^***^	0.59^**^	RMS+WL+ZC	0.85^***^	0.66^***^
WL	0.54^**^	0.60^***^	MAV+SSC+ZC	0.82^***^	0.87^***^
MAV+SSC	0.84^***^	0.81^***^	RMS+SSC+ZC	0.62^***^	0.89^***^
MAV+ZC	0.84^***^	0.79^***^	WL+SSC+ZC	0.88^***^	0.76^***^
RMS+SSC	0.65^***^	0.78^***^	RMS+WL+SSC+ZC	0.85^***^	0.90^***^
RMS+ZC	0.83^***^	0.84^***^	MAV+WL+SSC+ZC	0.91^***^	0.89^***^
WL+SSC	0.81^***^	0.84^***^	MAV+RMS+SSC+ZC	0.93^***^	0.92^***^
WL+ZC	0.80^***^	0.83^***^	MAV+RMS+WL+SSC	0.89^***^	0.82^***^
MAV+RMS+SSC	0.77^***^	0.74^***^	MAV+RMS+WL+ZC	0.89^***^	0.88^***^
MAV+RMS+ZC	0.87^***^	0.88^***^	MAV+ZC+SSC+RMS+WL	0.90^***^	0.93^***^

Significance levels are indicated as * for P ≤ 0.05,

** *for P ≤ 0.01, and*

*** *for P ≤ 0.001*.

*The bold values are the highest correlation coefficients achieved*.

### Performance Metrics

After the model configuration and feature selection, the mapping performances of the data-driven model were evaluated for both FMAs and MASs. The FMAs were further investigated with the sub-data sets for the pre-intervention and post-intervention sessions, respectively, since the sEMG patterns vary after the robot-assisted rehabilitation, as demonstrated in our previous clinical trials (Hu et al., 2013; Nam et al., 2017). It was possible that the performances for the two sub-sets of the data could differ; 80% of the sEMG epochs in a sub-set of data were assigned to the training set, and the rest were used as the testing set in the 5-fold cross validation. Moreover, mismatched tests (Li and Huang, 2010) were also investigated, i.e., using the post-intervention data sub-sets as the testing set for the BPNN trained by the pre-intervention data sub-sets, to further evaluate the heterogeneity between the two sub-sets of the data and the generalization of the data-driven model (Xu et al., 2013). The mismatched test might also have potential prognostic applications in the prediction of post-intervention effects based on pre-intervention status. We further evaluated the internal generalization (Brewer and Crano, 2000) with different proportions (i.e., 50%, 60%, 70%, 80%, and 90%) of the sEMG epochs from the post-intervention session as the training data, and then used the remained 50%, 40%, 30%, 20%, and 10% of the sEMG epochs as the respective testing data. Five-fold cross validation was used to train the data-driven model (i.e., 16 input nodes and 15 hidden nodes).

Additionally, the mapping performances were evaluated using sEMGs only from the proximal muscles, i.e., BIC and TRI, and the sEMGs only from the distal muscles, i.e., ED and FD, for the respective FMA-SE and FMA-WH. It was because that for unimpaired persons, the FMA-SE was mainly related to the function of the proximal muscles, e.g., BIC and TRI; while the FMA-WH was commonly associated with the function of the distal muscles, e.g., ED and FD (Fugl-Meyer et al., 1975; Qian et al., 2019). The BPNN was retrained when signals from only two muscles were used, and the number of input nodes was eight for the two sEMG channels. Bayesian regularization was employed to avoid the overfitting problem (Burden and Winkler, 2008), in case of the potential redundancy introduced by the reduction of input.

The mapping performance of the data-driven model from sEMG signals to the mapped MASs was first investigated by the correlation with different low-pass cutoff frequencies, i.e., 80, 150, 200, 300, 400, and 500 Hz, based on the MAS elbow scores for both the pre- and post-intervention datasets. Then, the cutoff frequencies that achieved the strongest correlation between the mapped MAS and the manual score were selected for further analysis of the mapping performances to the MAS elbow, wrist, and finger scores, using the data for the respective pre- and post-intervention sessions.

In our previous studies on robot-assisted upper limb rehabilitation (Hu et al., 2013; Nam et al., 2017; Qian et al., 2017; Huang et al., 2020), the sEMG parameters during the bare hand evaluation could reveal rehabilitative effects as observed in the clinical assessments, e.g., MAS and FMA. The sEMG parameters, i.e., activation level and CI, quantified the sEMG amplitude of a muscle and the timing of contraction between related muscle pairs. To compare the pre- and post-intervention sEMG patterns, sEMG activation level and CI, between a pair of muscles (Hu et al., 2009a), were further analyzed to quantify the variations in the sEMG patterns before and after the robot-assisted intervention.

The sEMG activation level for a muscle during the evaluation task was calculated as follows [and as defined previously (Hu et al., 2009a)]:

(7) $$\bar{\text{EMG}}=\frac{1}{T}\int_{0}^{T}EMG_{i}\left(t\right)dt,$$

where $\bar{\text{EMG}}$ refers to the average sEMG envelope value of muscle *i*. *EMG*_*i*_(*t*) is the sEMG envelope signal obtained after normalization with respect to the maximal value of the muscle in the session, and *T* is the length of the signal trial. A decrease in the sEMG amplitude measured by the activation level of a muscle after rehabilitation could indicate the release of muscular spasticity (Hu et al., 2009a).

The CI between a pair of muscles is expressed as follows:

(8) $$CI=\frac{1}{T}\int_{0}^{T}A_{ij}\left(t\right)dt,$$

where *A*_*ij*_*(t)* is the overlapping activity of sEMG linear envelopes for muscles *i* and *j*, and *T* is the length of the signal. An increase in the CI value represented increased co-contraction of a muscle pair (broadened overlapping area), and a decrease in the CI value indicated decreased co-contraction of a muscle pair (reduced overlapping area), i.e., better co-ordination (Hu et al., 2009a). The sEMG activation level and CI were adopted as secondary outcomes in the work.

The normalized sEMG parameters (Nam et al., 2017) for the pre- and post-intervention sessions were compared by the paired *t*-test, after the normality tests on the sEMG samples by the Shapiro–Wilk test, with a significance level of 0.05. All sEMG parameters satisfied the normal distribution. The same normality tests on the clinical scales for the pre- and post-intervention sessions were also performed. The FMA-WH and FMA-SE data followed normal distributions (*P* > 0.05), while the MASs did not. The comparisons between pre- and post-intervention sessions of the FMAs and MASs were evaluated by the paired *t*-test and Wilcoxon test, respectively. The level of statistical significance was set at 0.05, and significance levels at 0.01 and 0.001 were also indicated in the Results section.

## Results

### Model Configuration and Feature Selection

Table 2 summarizes the significant correlation coefficients between the manual FMA subscores and the mapped scores obtained by BPNN with different numbers of hidden nodes. The strongest correlation was found with the hidden layer of 15 nodes (*r* = 0.90 for FMA-SE and *r* = 0.93 for FMA-WH, *P* < 0.001), and the correlation coefficients decreased with the number of hidden nodes exceeding or less than 15. Table 3 shows the mapping performance of the model with different input feature vectors by the combinations of the sEMG amplitude features and those related to the MU firing. The highest correlation between the mapped scores and the manual scores, i.e., *r* = 0.93 for FMA-SE and *r* = 0.92 for FMA-WH, *P* < 0.001, was observed in the combination of MAV, SSC, RMS, and ZC. All feature combinations produced significant correlations (*P* < 0.01) between the mapped and the manual FMA subscores, and the minimum *r* was 0.54 (for FMA-SE, *P* < 0.01) when using only WL as the input feature. As the number of features in a combination increased, the correlation between the mapped and the manual FMA subscores was also enhanced. However, the combination of all five features led to a decrement of 0.3 and an increment of 0.1 on the correlation coefficient between the mapped and the manual FMA-SE and FMA-WH scores, respectively, compared to the maximum coefficient achieved by the combination of MAV, SSC, RMS, and ZC.

### Mapping Performance for FMA and MAS

**Figure 3:**
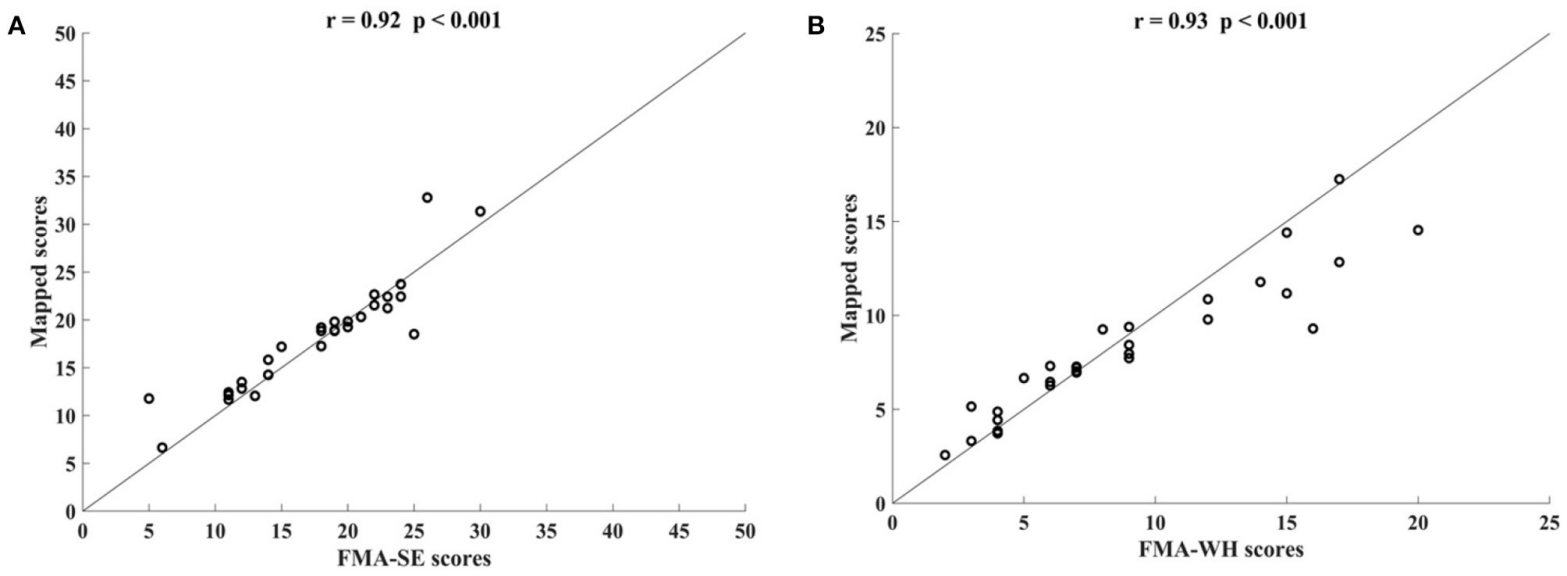
Significant correlations yielded by the four-channel sEMG signals, i.e., ED, FD, BIC, and TRI, before the 20-session intervention. Correlations between the mapped scores and manual **(A)** Fugl–Meyer Assessment shoulder/elbow (FMA-SE) scores and **(B)** Fugl–Meyer Assessment wrist/hand (FMA-WH) scores.

**Figure 4:**
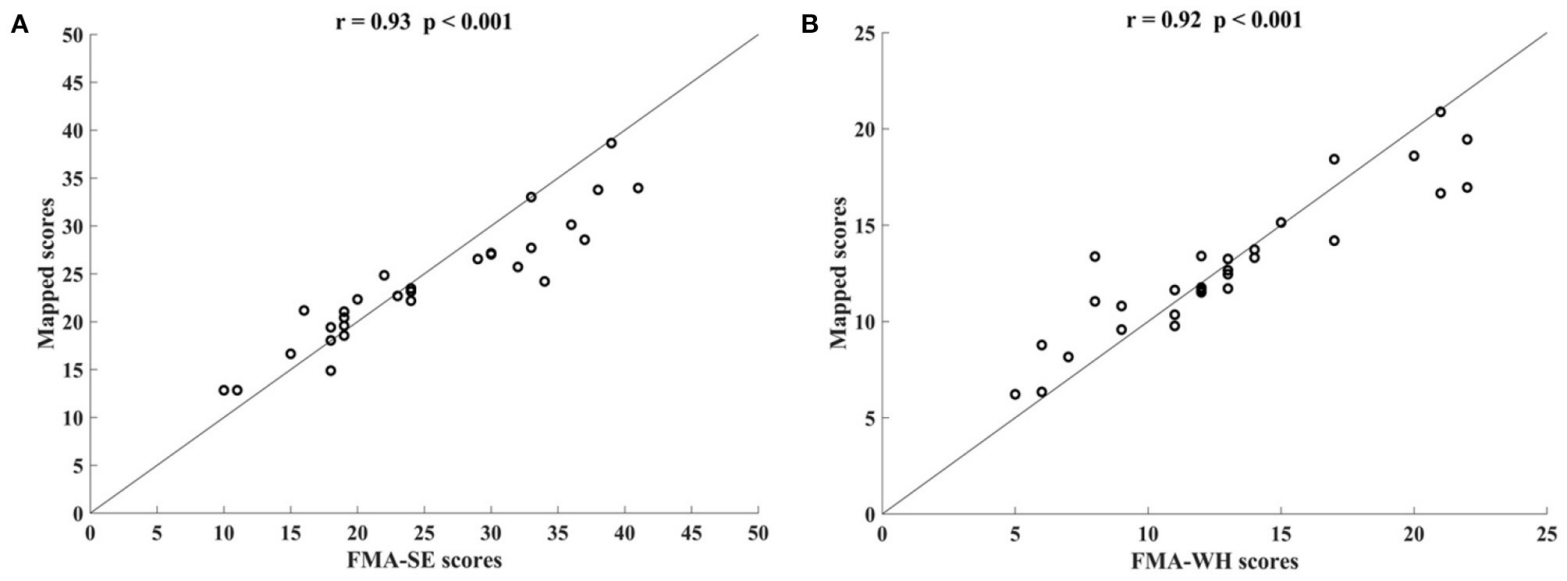
Significant correlations yielded by the four-channel sEMG, i.e., ED, FD, BIC, and TRI, after the 20-session intervention. Correlations between the mapped scores and manual **(A)** Fugl–Meyer Assessment shoulder/elbow (FMA-SE) scores and **(B)** Fugl–Meyer Assessment wrist/hand (FMA-WH) scores.

Figure 3 shows the correlations between the mapped scores with the manual FMA subscores before the 20-session intervention. Significant correlations between the mapped and the manual scores were observed (*r* = 0.92 for FMA-SE, and *r* = 0.93 for FMA-WH, *P* < 0.001). The mapped FMA-SE scores ranged from 6.65 to 32.80 when the manual scores ranged from 5 to 30; the mapped FMA-WH scores ranged from 2.57 to 17.25 when the manual scores ranged from 2 to 20. The mapped scores were closer to the manual ones when the manual FMA-SE and FMA-WH scores were 11–24 and 2–9, respectively, than within other manual FMA-SE and FMA-WH score distributions. Figure 4 shows the correlations between the mapped and the manual FMA subscores after the 20-session intervention. The resultant mapped scores were positively correlated with the manual FMA-SE (*r* = 0.93, *P* < 0.001) and FMA-WH scores (*r* = 0.92, *P* < 0.001). The mapped FMA-SE scores ranged from 12.84 to 39 when the manual scores ranged from 10 to 41; the mapped FMA-WH scores ranged from 6.22 to 21 when the manual scores ranged from 5 to 22. The mapped scores were the closest to the manual ones for manual FMA-SE and FMA-WH scores between 15–24 and 9–15 points, respectively. For both the pre- and post-intervention datasets, the correlation between the mapped FMA subscores and the manual scores were numerically high.

**Figure 5:**
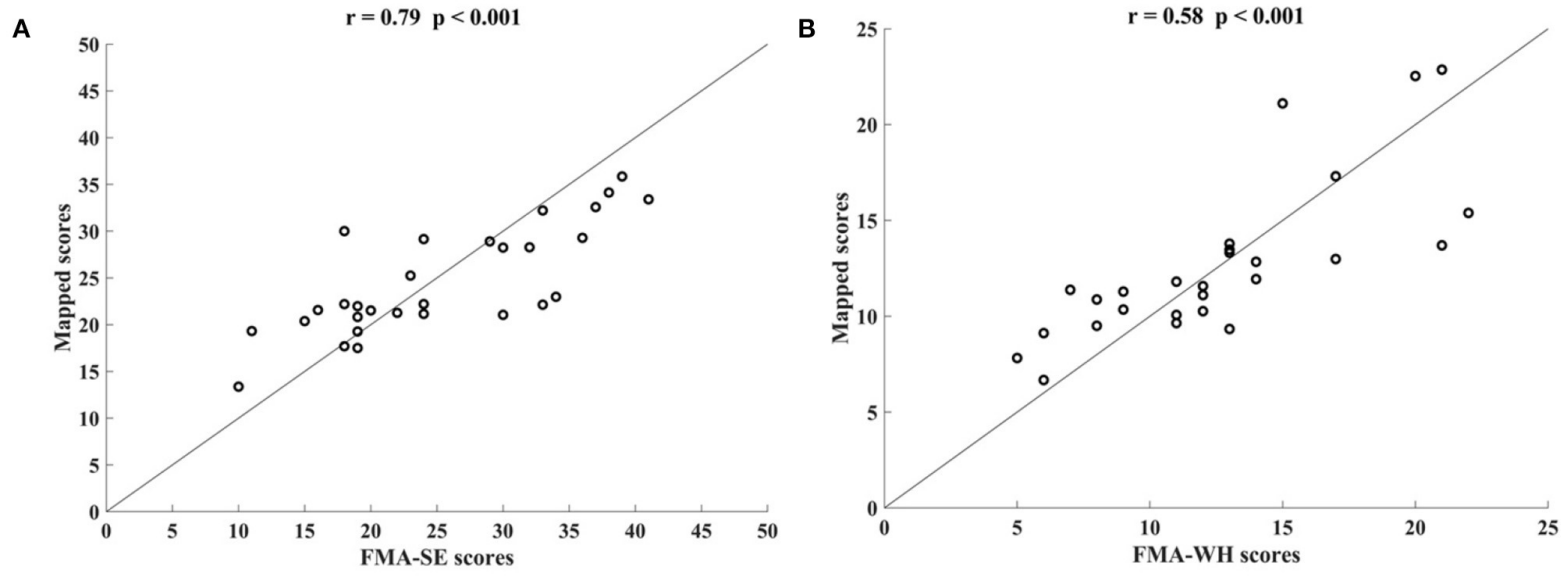
Significant correlations yielded by the two-channel sEMG signals after the 20-session intervention. Correlation between the mapped scores and manual **(A)** Fugl–Meyer Assessment shoulder/elbow (FMA-SE) scores, from muscles pair of BIC and TRI, **(B)** Fugl–Meyer Assessment wrist/hand (FMA-WH) scores, from muscle pair of ED and FD.

**Figure 6:**
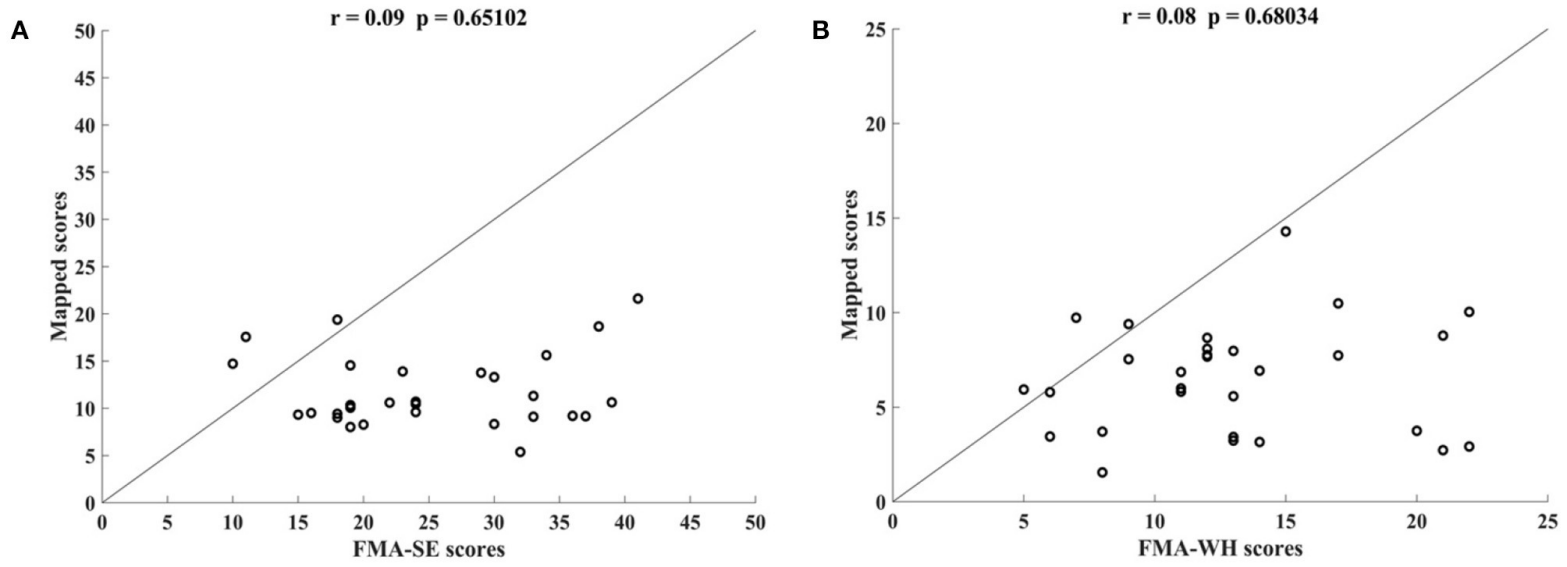
Correlations yielded by the mismatched testing condition with the four-channel sEMG signals, i.e., ED, FD, BIC, and TRI. Correlation between the mapped scores and manual **(A)** Fugl–Meyer Assessment shoulder/elbow (FMA-SE) scores and **(B)** Fugl–Meyer Assessment wrist/hand (FMA-WH) scores.

Figure 5 shows the correlations between the manual FMA subscores and the mapped scores using sEMG signals from the two corresponding muscles, i.e., BIC and TRI for FMA-SE; ED and FD for FMA-WH, after the 20-session intervention. The correlation coefficients between the mapped scores and the manual scores were 0.79 for FMA-SE (*P* < 0.001) and 0.58 for FMA-WH (*P* < 0.001), respectively. The mapped FMA-SE and FMA-WH scores ranged from 13.37 to 35.85 and from 6.67 to 22.87, respectively. The distributions of manual scores were the same as those in Figure 4. The correlation coefficient between the mapped and manual FMA-SE scores was higher than that of the FMA-WH scores. Figure 6 shows the correlations between the manual and mapped FMA subscores obtained under the mismatched testing condition after the intervention. No significant correlation was found between the mapped scores and the manual scores of FMA-SE and FMA-WH. The mapped FMA-SE and FMA-WH scores ranged from 5.38 to 21.62 and from 1.55 to 14.29, respectively; the manual FMA-SE and FMA-WH scores ranged from 10 to 41 and from 5 to 22, respectively. For both FMA-SE and FMA-WH, the mapped scores were generally lower than the manual scores under the mismatched testing condition after the intervention. Table 4 shows the correlation between the mapped and manual FMA subscores, by the BPNN model with different proportions of the training data in the internal generalization evaluation. The correlation maintained above 0.88 when the proportion of training data reduced to 50%, i.e., split-half method (Steyerberg et al., 2001).

**Table 4:** The correlation coefficients, *r*, between the mapped and manual FMA scores, obtained by the BPNN model trained with different distributed proportions of the training and testing data.

**Proportion of training data**	***r* with FMA-SE**	***r* with FMA-WH**
50%	0.89^***^	0.88^***^
60%	0.91^***^	0.90^***^
70%	0.89^***^	0.92^***^
80%	0.93^***^	0.92^***^
90%	0.87^***^	0.88^***^

Significance levels are indicated as* for P ≤ 0.05, ** for P ≤ 0.01, and

*** *for P ≤ 0.001*.

**Figure 7:**
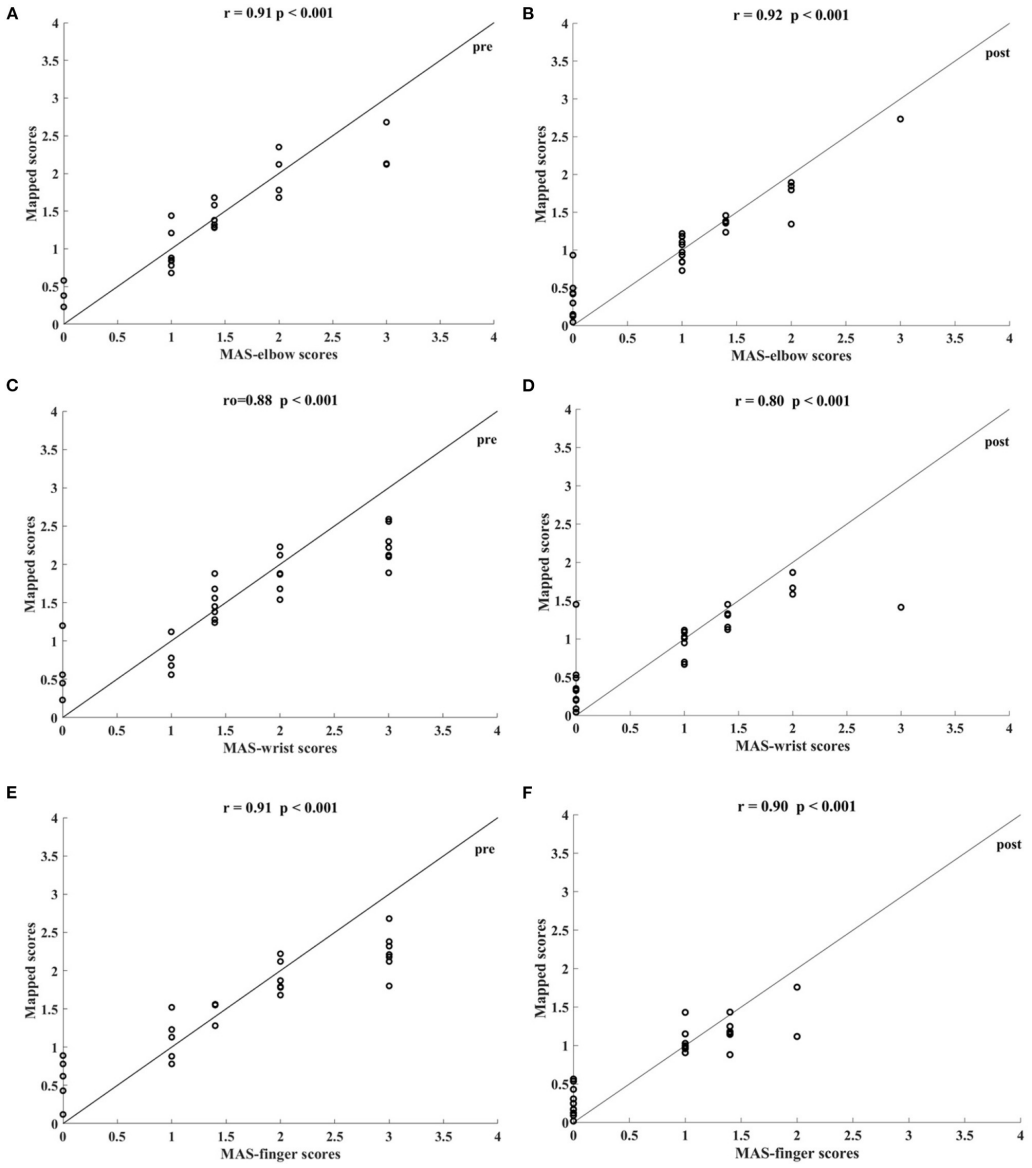
Significant correlations between the mapped scores and MASs scales yielded by the bandpass filtered (10–200 Hz) four-channel sEMG signals, i.e., ED, FD, BIC, and TRI. The correlations between the mapped scores and MASs **(A)** at elbow for the pre-intervention dataset, **(B)** at elbow for the post-intervention dataset, **(C)** at wrist for the pre-intervention dataset, **(D)** at wrist for the post-intervention dataset, **(E)** at fingers for the pre-intervention dataset, and **(F)** at fingers for the post-intervention dataset.

Table 5 shows the correlation coefficients between the manual and the mapped MAS-elbow scores obtained using the sEMG signals with different lowpass cutoff frequencies. All correlations were statistically significant (*P* < 0.05). The correlation coefficient increased with increasing lowpass cutoff frequency and reached a maximum of 0.92 (*P* < 0.001) when the cutoff frequency was 200 Hz. The correlation decreased when the cutoff frequency was greater than 200 Hz. Figure 7 shows the correlations between the manual and mapped MAS scores obtained for the 10–200 Hz band-pass filtered sEMG data. Significant correlations were observed between the mapped scores and the manual MASs for both the pre- and post-intervention datasets. For the pre-intervention dataset, the correlations between the mapped and manual scores were 0.91 (*P* < 0.001, MAS-elbow), 0.88 (*P* < 0.001, MAS-wrist), and 0.91 (*P* < 0.001, MAS-finger). For the post-intervention dataset, the correlations between the mapped and manual scores were 0.92 (*P* < 0.001, MAS-elbow), 0.80 (*P* < 0.001, MAS-wrist), and 0.90 (*P* < 0.001, MAS-finger). The correlation coefficient between the mapped and the manual MAS-wrist scores observed in the pre-intervention data was larger than that of the post-intervention data. For the pre-intervention dataset, the distances between the mapped and manual MAS scores exceeded 0.5 when the manual MASs were graded at 0 and 3. For the post-intervention data, the distances between the mapped and manual MAS scores exceeded 0.5 when the manual MASs were graded at 0, 2, and 3.

**Table 5:** The correlation coefficients, *r*, between the mapped and manual MAS-elbow scores and the scores obtained by the sEMG signals with different lowpass cutoff frequencies.

**Cutoff frequency (Hz)**	***r* with MAS-elbow**
80	0.76^***^
150	0.89^***^
200	0.92^***^
300	0.81^***^
400	0.75^***^
500	0.42^***^

Significance levels are indicated as* for P ≤ 0.05, ** for P ≤ 0.01, and

*** *for P ≤ 0.001*.

### sEMG Parameters and Clinical Scales Before and After the Intervention

**Figure 8:**
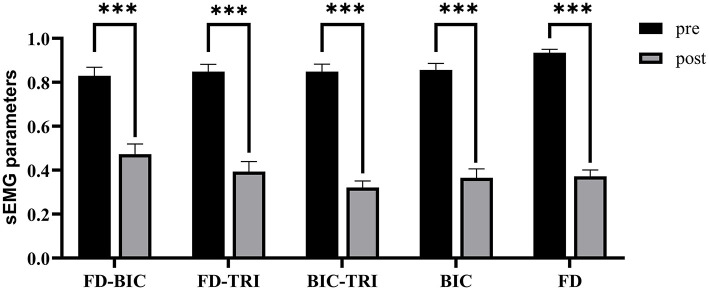
The differences of sEMG parameters between the pre- and post-intervention sessions. The x-axis indicates the target muscles (BIC and FD) and muscle pairs (FD-BIC, FD-TRI, and BIC-TRI). The y-axis indicates the corresponding CI of the muscle pairs and the normalized sEMG activation level at the target muscles. The significant differences are indicated as * for *P* ≤ 0.05, ** for *P* ≤ 0.01, and ***for *P* ≤ 0.001, using the paired *t*-test.

**Figure 9:**
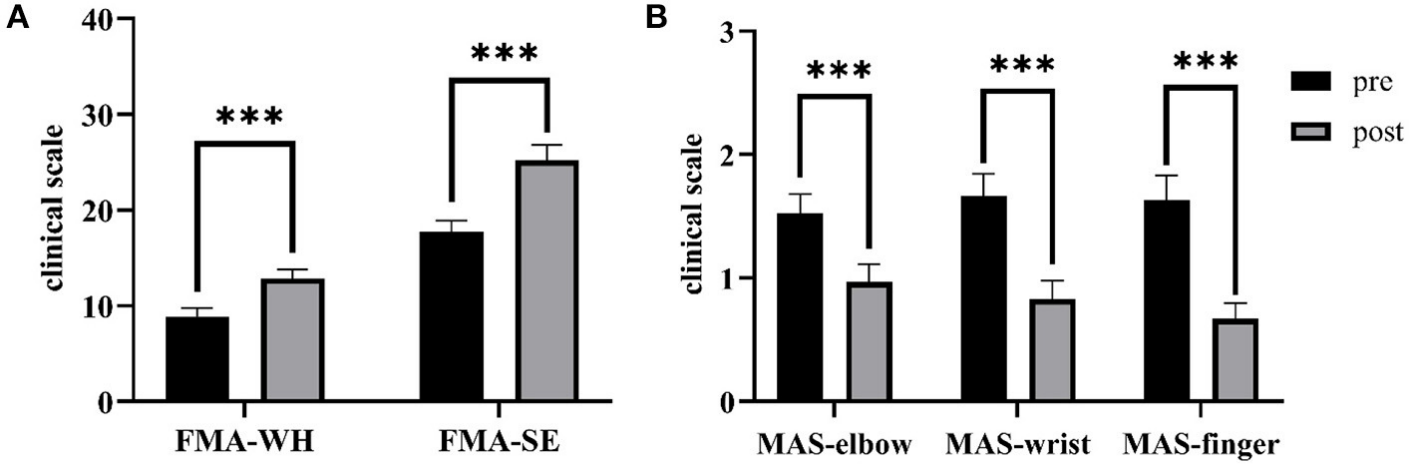
The differences of clinical scales between the pre- and post-intervention sessions. **(A)** Sub-FMA scores, **(B)** MASs. The significant differences are indicated as * for *P* ≤ 0.05, ** for *P* ≤ 0.01, and ***for *P* ≤ 0.001, using the paired *t*-test for the FMA and the Wilcoxon test for the MAS.

Figure 8 shows the comparisons between the two sEMG parameters, i.e., the normalized sEMG activation level and the normalized CI, for the pre- and post-intervention sessions. Significant differences in sEMG activation level were observed at the BIC and FD (paired *t*-test, *P* < 0.05) muscles, and the activation levels of the BIC and FD muscles before the intervention were higher than those after the intervention. There were significant differences in the CI of the FD-BIC, FD-TRI, and BIC-TRI muscle pairs (paired *t*-test, *P* < 0.05) before and after the interventions. The CI values detected before the intervention were higher than those after the interventions. No significant difference was observed in the sEMG parameters of other target muscles and muscle pairs. Table 6A presents the differences in the sEMG parameters between the pre- and post-intervention sessions. Figure 9 shows the pre- and post-intervention comparisons of the clinical scales. Significant differences in the FMA subscores, i.e., FMA-SE and FMA-WH (paired *t*-test, *P* < 0.05), and MAS scores, i.e., MAS-elbow, MAS-wrist, and MAS-finger (Wilcoxon test, *P* < 0.05), were observed between the pre- and post-intervention sessions. The pre-intervention FMA-WH and FMA-SE scores were lower than the post-intervention scores. The MAS-elbow, MAS-wrist, and MAS-finger scores before the intervention were higher than those obtained after the intervention. The differences in clinical scales before and after intervention are shown in Table 6B.

**TABLE 6A:** The differences in the sEMG parameters between the pre- and post-intervention sessions.

	**Pre-intervention**	**Post-intervention**	**P**
	**Mean(± std)**	**Mean(± std)**	
BIC	0.86 (± 0.14)	0.36 (± 0.19)	0.000^***^
FD	0.93 (± 0.07)	0.37 (± 0.13)	0.000^***^
FD-BIC	0.83 (± 0.18)	0.47 (± 0.21)	0.000^***^
FD-TRI	0.85 (± 0.15)	0.39 (± 0.21)	0.000^***^
BIC-TRI	0.85 (± 0.16)	0.32 (± 0.13)	0.000^***^

BIC and FD indicate the normalized sEMG activation level at the target muscles. FD-BIC, FD-TRI, and BIC-TRI indicate the normalized CI of the muscle pairs. Significance levels of the paired t-test are indicated as * for P ≤ 0.05, ** for P ≤ 0.01, and

*** *for P ≤ 0.001*.

**TABLE 6B:** The differences in the clinical scales between the pre- and post-intervention sessions.

	**Pre-intervention**	**Post-intervention**	**P**
	**Mean(±std)**	**Mean(±std)**	
FMA-WH	8.86 (± 2.49)	12.90 (± 2.45)	0.000^***^
FMA-SE	17.76 (± 3.03)	25.21 (± 4.37)	0.000^***^
MAS-elbow	1.52 (± 0.42)	0.97 (± 0.39)	0.001^***^
MAS-wrist	1.66 (± 0.48)	0.83 (± 0.41)	0.000^***^
MAS-finger	1.63 (± 0.55)	0.67 (± 0.35)	0.000^***^

The FMA-WH and FMA-SE are compared by using the paired t-test. The MAS-elbow, MAS-wrist, and MAS-finger scores are compared by using the Wilcoxon test. Significance levels of the paired t-test and Wilcoxon test are indicated as * for P ≤ 0.05, ** for P ≤ 0.01, and

*** *for P ≤ 0.001*.

## Discussion

This study developed an sEMG data-driven BPNN model for mapping sEMG signals to two clinical scales, FMA subscores (FMA-SE and FMA-WH) and MAS, that have been widely adopted in clinical practices in post-stroke rehabilitation. The model was optimized by changing the number of the nodes in the hidden layer of the BPNN and the input feature vectors. The mapped FMA subscores and MAS scores generated by the data-driven model were strongly correlated with the manual scores (*r* > 0.9, Figures 3, 4, 7) (Evans, 1996) for both the pre- and post-intervention datasets.

### Model Configuration

In this work, the sEMG data-driven model was established based on a three-layer BPNN. Using the two-phase method to determine the number of hidden layer neurons, the performances of the models with different numbers of nodes (10, 15, 20, 30, 40, 50, 100, and 200) in the hidden layer were evaluated. All models yielded high (i.e., *r* > 0.8, Table 2) correlation coefficients between the mapped scores and the manual scores, which demonstrates the three-layer BPNN is feasible and sufficient for continuous mapping of the sEMG data to the clinical scales of FMA subscores (De Villiers and Barnard, 1993; Freedman, 2009). The BPNN with 15 nodes in the hidden layer performed the best when mapping sEMG data to the clinical scales of FMA subscores, generating the highest correlation coefficient between the mapped and the manual scores. The number of hidden nodes, i.e., 15, was 75 percent of the dimensionality of the input layer i.e., 20, according to the rule of the thumb method, which suggested using 70% to 90% of the size of the input layer as the number of hidden nodes (Boger and Guterman, 1997). The relatively poor performances of models with more than 15 hidden nodes were caused by model overfitting. The overfitting BPNN model with excessive hidden nodes could create complex fitting between the unrepresentative characteristics of the sEMG training dataset and the manual scores, which reduced the generalization accuracy in the testing dataset (Sheela and Deepa, 2013). On the other hand, the relatively poor performance of models with less than 15 hidden nodes could be due to model underfitting. The model containing insufficient hidden nodes lacked flexibility in terms of linear regression, decreasing its accuracy for the testing dataset (Mitchell, 1997).

### Input Feature Selection

Different combinations of the five features of the sEMG signals (MAV, SSC, RMS, ZC, and WL) served as the input vectors to the BPNN model (Table 3). All features alone could produce moderate or higher correlations (Evans, 1996), i.e., *r* ≥ 0.54, between the mapped scores and the manual FMA subscores. This indicated that these features contained the representative neuromuscular information of motor functional recovery during robot-assisted rehabilitation, which could be recognized by the BPNN data-driven model of this work. The performance of the models improved if the feature contained both magnitude-related features (MAV, RMS, and WL) and MU-firing-related features (ZC and SSC), which demonstrates that both the magnitude and MU firing information of the sEMG signals are essential for mapping performance. In addition, the performance of the models improved as the number of features in a combination increased. However, instead of the full combination of all five features, the combination of MAV, SSC, RMS, and ZC without WL produced the highest correlation coefficient between the mapped and manual scores, suggesting that WL might be a redundant feature that weakened mapping performance. Furthermore, WL alone yielded the poorest performance (*r* = 0.54) among all the combinations, also indicating that WL was not an effective feature for mapping the sEMG signals to the clinical scales of FMA. Therefore, in this work, the combination of MAV, SSC, RMS, and ZC was selected as the input vector for the BPNN. This optimized feature combination was consistent with a previous study of feature reduction and selection for sEMG signal classification (Phinyomark et al., 2012).

### Mapping the sEMG Data to FMA Subscores

In this study, the sEMG signals from four muscles, i.e., BIC, TRI, ED, and FD, were mapped to FMA-SE and FMA-WH subscores. Significant correlations (*r* > 0.90) were observed between the mapped and manual FMA subscores, i.e., the FMA-SE and FMA-WH, before and after the intervention (Figures 3, 4). Therefore, the mapped scores were highly consistent with the manual scores of FMA for both the pre- and post-intervention sessions. Furthermore, the best regression performance occurred when the manual scores were around their mean values (e.g., 17.76 in Figure 3A). This was because the manual FMA subscores in the training dataset followed a normal distribution (e.g., μ = 17.76, σ = 3.03 in Figure 3A) and the training data were concentrated around the mean values. When the manual scores were far away from the mean value, there were insufficient training data for the model to learn the mapping relationship between the sEMG data and the FMA subscores.

In addition, the sEMG from two corresponding muscle pairs, i.e., BIC and TRI for proximal movements; ED and FD for distal movements, were mapped to manual scores of FMA-SE and FMA-WH, respectively. The correlations between the mapped and manual scores produced by the muscle pairs were lower than those produced by the four muscles (Figure 5). This demonstrates that the compensatory muscular activities, especially proximal compensations, play a critical role in the bare hand evaluation task during robot-assisted rehabilitation in chronic stroke. Proximal muscular compensation was observed more frequently than distal compensation during the evaluation of motor functional recovery. Therefore, the correlation between the mapped and manual FMA-WH scores produced by the distal muscle pair was much lower than that between the mapped and manual FMA-SE scores produced by the proximal muscle pair (Figure 4).

### Generalization of the Model

The performance of generalization of the data-driven model was tested under the mismatched testing condition, where the model was trained by the dataset before the intervention and tested by the dataset after the intervention. No significant correlations were observed between the mapped and the manual scores of FMA-SE and FMA-WH (Figure 6), demonstrating that the model learned from the training data before the intervention and could not map the sEMG data to the manual FMA subscores after the intervention. Table 6A; Figure 8 show the differences between the analyzed sEMG parameters, i.e., the normalized sEMG activation level and CI, before and after the intervention, which explained the results of the mismatched testing condition. In addition, the correlation maintained above 0.88 even when the proportion of training data reduced to 50% (Table 4), which suggested that the model demonstrated a satisfying internal generalization with limited samples, i.e., half of the limited training sets (Steyerberg and Harrell, 2016).

For both the FMA-WH and FMA-SE, the mapped scores were generally lower than the manual scores (Figure 6), which indicated that the model underestimated motor function recovery after the intervention. This was because the model was trained by the data obtained before the intervention, when the manual FMA subscores were significantly lower than those after the intervention (Table 6B; Figure 9). This suggested that although the current data-driven model could map the sEMG data to the manual FMA subscores either before or after intervention in chronic stroke with stable muscular activities pattern, it was not able to predict the manual FMA subscores after the intervention in a prognostic way.

### Mapping the sEMG Data to MAS

Besides mapping to the FMA scores, the data-driven model was also used to map the sEMG data to clinical scales of the MAS. For mapping to the MAS, sEMG signals were low-pass filtered and the cutoff frequency was set according to the characteristics of sEMG and previous studies on involuntary contractions related to spasticity (Sahrmann and Norton, 1977; Van Boxtel, 2001). The best mapping performance occurred when the sEMG signals were low-pass filtered at 200 Hz (Table 5), demonstrating that the low-frequency component of sEMG signals effectively reflected the slow involuntary contractures in the spastic muscle after stroke (Dromerick, 2002). Compared to the cutoff frequencies of 150 and 300 Hz, the correlation reached a peak value when the low-pass cutoff frequency was 200 Hz. This suggested that the most effective sEMG frequency in the spastic muscle exists around 200 Hz. This result further suggested that the motor units in the spastic muscles mainly show low-frequency firing, which was consistent with previous findings (Sahrmann and Norton, 1977). Further, the correlation between the mapped and manual MAS-elbow scores decreased when the sEMG was filtered at frequencies lower than 200 Hz (Table 5), indicating that the much lower-frequency sEMG signals lost bio-information related to muscle spasticity.

Significant correlations between the mapped and manual MASs (Figure 7) indicated that the sEMG data-driven model can map the sEMG data to MAS, and the high consistency of the mapped scores could aid in the diagnosis of muscle spasticity among chronic stroke survivors. However, this data-driven model did not perform well when the MAS grades at 0 and 3, i.e., the distances between the mapped and manual scores exceeded 0.5. This was because the recruitment criteria required the MASs of participants to be lower than 3 and above zero and restricted the model to learning from the limited training data to the mapped MAS of 0 and 3. This phenomenon was also noted in previous studies (Zhang et al., 2019; Yu et al., 2020). Moreover, the correlation between the mapped and manual MAS-wrist scores observed in the dataset before the intervention was higher than those observed after the intervention. This was because a more scattered distribution of the training data produced better mapping from the sEMG data to the clinical scales of MAS, while the manual MASs decreased significantly after the intervention (Table 6B; Figure 9) and were concentrated among the low scores.

There were limitations in this study. The generalization was unsatisfied under the mismatched testing condition. The possible reasons were as follows: (1) The limited sample size of the recruited subjects (*n* = 29) hindered the model's effective generalization, i.e., recognizing completely new inputs, compared to those achieved this in the literatures usually with larger sample sizes (e.g., *n* > 100) (Kim et al., 2020; Scrutinio et al., 2020). 2) There were significant variations in sEMG properties and clinical scores after the 20-session robot-assisted rehabilitation for the participants, as shown in Tables 6A,B; Figures 8, 9. The sEMG patterns and the clinical scores after the robot-assisted intervention could be regarded as new inputs to the data-driven model, as revealed by the insignificant correlations in the mismatched test. In our future work, more participants will be recruited for the collection of independent clinical scores and sEMG trials, to achieve better generalization, not only on the post-training data, but also on data of new participants. In addition, sEMG parameters (e.g., the CI and activation level) and clinical diagnostic information will be involved to improve the robustness of the features in the future work. In order to facilitate the clinical translation in the future, explanation to clinical practitioners would be carried out on the working principle of the BPNN, i.e., non-linear mapping, in contrast to the linear regressions, i.e., linear mapping, in the traditional explainable methods.

## Conclusion

This study presented an sEMG data-driven BPNN model for mapping sEMG data in bare hand daily tasks to two widely used clinical scales, the FMA and the MAS. The combination of four features of sEMG (MAV, RMS, SSC, and ZC) as the input vector into the BPNN model optimized the mapping accuracy. The high correlations between the mapped scores and the manual MAS and FMA subscores suggested that the sEMG data-driven BPNN model could evaluate upper limb motor functions based on sEMG signals. The results demonstrated the potential application in automated assessment without close professional operation, or supervision, by a clinician as in the evaluation of FMA and MAS for chronic stroke, once the external generalization of the model could be validated with large sample sizes.

## Data Availability Statement

The original contributions presented in the study are included in the article/supplementary material, further inquiries can be directed to the corresponding authors.

## Ethics Statement

The studies involving human participants were reviewed and approved by Human Subjects Ethics Committee of Hong Kong Polytechnic University (HSEARS20130306005-04 and CRE-2013.283-T). The patients/participants provided their written informed consent to participate in this study.

## Author Contributions

FY contributed to the construction of the data-driven BPNN model, data analysis, and manuscript drafting. BY contributed to the manuscript drafting. CN and YX contributed to the robotic-hand-assisted training experiment and data collection. XH and FC conceived of the study and coordinated the whole project, including the experiment design, model construction, and manuscript drafting. All authors contributed to the article and approved the submitted version.

## Conflict of Interest

The authors declare that the research was conducted in the absence of any commercial or financial relationships that could be construed as a potential conflict of interest.
